# The association of coronary artery calcium progression and cognitive function in midlife ‐ The Coronary Artery Risk Development In Young Adults (CARDIA) Study

**DOI:** 10.1002/alz.089981

**Published:** 2025-01-09

**Authors:** Masayuki Teramoto, Tina D Hoang, Stephen Sidney, James G. Terry, Mark J Pletcher, Gregory M Marcus, Thomas Hoffman, Kristine Yaffe

**Affiliations:** ^1^ Departments of Epidemiology and Biostatistics, University of California San Francisco, San Francisco, CA USA; ^2^ NCIRE‐The Veterans Health Research Institute, San Francisco, CA USA; ^3^ Kaiser Permanente of Northern California, Oakland, CA USA; ^4^ Vanderbilt University Medical Center, Nashville, TN USA; ^5^ Department of Epidemiology & Biostatistics, University of California San Francisco, San Francisco, CA USA; ^6^ Departments of Cardiology, University of California San Francisco, San Francisco, CA USA; ^7^ University of California San Francisco, San Francisco, CA USA; ^8^ Department of Psychiatry and Behavioral Sciences, University of California, San Francisco, San Francisco, CA USA; ^9^ Department of Epidemiology, University of California, San Francisco, San Francisco, CA USA

## Abstract

**Background:**

Little is known about the impact of coronary artery calcium (CAC) and its progression on cognitive function in midlife, a time of importance for cognitive aging.

**Methods:**

In CARDIA, CAC was measured using computed tomography at years 15 (baseline), 20 and 25. CAC progression was defined as: (1) CAC>0 at follow‐up among participants with baseline CAC = 0; (2) an annualized change of ≥10 units at follow‐up among those with 0<baseline CAC<100; or (3) an annualized percent change (annualized change in CAC score divided by baseline CAC score) ≥10% among those with baseline CAC≥100. Cognitive function was measured with Digit Symbol Substitution Test (DSST), Rey Auditory Verbal Learning Test (RAVLT), Stroop Interference, Montreal Cognitive Assessment (MoCA) and Verbal Fluency (Letter and Category Fluency) at year 35. We used linear regression to estimate the difference between marginal means with 95% confidence intervals (CIs) of each cognitive test by CAC score (0 vs >0) and CAC progression.

**Results:**

Among 2,341 participants (mean age 40.3 ± 3.6 years, 56% female, 43% Black at baseline), higher CAC score and CAC progression were associated with significantly lower cognitive performance in all cognitive tests. After adjustment for demographics, the association remained for CAC progression, compared to no progression, for most test scores: DSST (‐2.70 95% CI: ‐4.05 to ‐1.36, p < 0.001), MoCA (‐0.44 95% CI: ‐0.74 to ‐0.13, p = 0.005) and verbal fluency (‐0.85 95% CI: ‐1.57 to ‐0.12, p = 0.02). Similar associations were observed for higher CAC score (>0 vs 0): DSST (‐3.11 95% CI: ‐5.16 to ‐1.06, p = 0.003), MoCA (‐0.81 95% CI: ‐1.28 to ‐0.34, p = 0.001) and RAVLT (‐0.67 95% CI: ‐1.12 to ‐0.23, p = 0.003). Additional adjustment for physical activity, depressive symptoms, and APOE, and mutually for baseline CAC score and progression did not materially change the association.

**Conclusions:**

CAC score and progression were associated with lower cognitive function in midlife. CAC progression may be an independent risk factor for early cognitive aging, independent of baseline CAC score and other risk factors.

